# Supplementary material for: A portable regulatory RNA array design enables tunable and complex regulation across diverse bacteria
Source: Nat Commun. 2023 Aug 29;14:5268. doi: 10.1038/s41467-023-40785-x (PMC10465534; doi:10.1038/s41467-023-40785-x)
Supplement: Supplementary file 4 — Description of Additional Supplementary Files [file 41467_2023_40785_MOESM4_ESM.pdf]

Title: Supplementary Data 1.
Description: Genebank files for plasmid maps used in this study.

Title: Supplementary Data 2.
Description: Sequencing data for experiments shown in Supplementary Figure 10.
